# Safety profile of preoperative administration of low-molecular-weight heparin on minimally invasive lung cancer surgery: a randomized controlled trial

**DOI:** 10.1186/s12893-021-01244-w

**Published:** 2021-05-19

**Authors:** Gu-Ha A-Lai, Ze-Guo Zhuo, Gang Li, Tie-Niu Song, Zhi-Jie Xu, Xu Shen, Peng Yao, Yi-Dan Lin

**Affiliations:** 1 Department of Thoracic Surgery, West China Hospital, Sichuan University, No. 37 Guoxue Alley, Chengdu, 610041 China; 2 Department of Thoracic Surgery, Chengdu Office Hospital Affiliated Tibet Autonomous Region, Chengdu, 610041 China

**Keywords:** Lung cancer, Low-molecular-weight heparin, Minimally invasive surgery, Thromboprophylaxis

## Abstract

**Background:**

Venous thromboembolism remains a common but preventable complication for cancerous lung surgical patients. Current guidelines recommend thromboprophylaxis for lung patients at high risk of thrombosis, while a consensus about specific administration time is not reached. This study was designed to investigate the safety profile of preoperative administration of low-molecular-weight-heparin (LMWH) for lung cancer patients.

**Methods:**

From July 2017 to June 2018, patients prepared to undergo lung cancer surgery were randomly divided into the preoperative LMWH-administration group (PRL) for 4000 IU per day and the postoperative LMWH-administration group (POL) with same dosage, all the patients received thromboprophylaxis until discharge. Baseline characteristics including demographics and preoperative coagulation parameters were analyzed, while the endpoints included postoperative coagulation parameters, postoperative drainage data, hematologic data, intraoperative bleeding volume and reoperation rate.

**Results:**

A total of 246 patients were collected in this RCT, 34 patients were excluded according to exclusion criterion, 101 patients were assigned to PRL group and 111 patients belonged to POL group for analysis finally. The baseline characteristic and preoperative coagulation parameters were all comparable except the PRL group cost more operation time (p = 0.008) and preoperative administration duration was significantly longer (p < 0.001). The endpoints including postoperative day 1 coagulation parameters, mean and total drainage volume, drainage duration, intraoperative bleeding volume and reoperation rate were all similar between the two groups. Moreover, coagulation parameters for postoperative day 3 between the two groups demonstrated no difference.

**Conclusion:**

Preoperative administration of low-molecular-weight-heparin demonstrated safety and feasibility for lung cancer patients intended to receive minimally invasive surgery.

*Trial registration:* ChiCTR2000040547 (www.chictr.org.cn), 2020/12/1, retrospectively registered.

## Background

Lung cancer is the most common malignancy and the first cause of cancer death worldwide, with more than 2 million new diagnosis and around 1.8 million death in 2018 [1]. Venous thromboembolism (VTE) contains deep vein thrombosis (DVT) and pulmonary embolism (PE). And VTE could result in higher mortality and morbidity, longer hospital duration, higher in-hospital cost and poorer life quality [2, 3]_._ Previous survey of symptomatic VTE found more than 460 thousand cases of DVT and about 300 thousand cases of PE per annum in European Union, while the estimated VTE-related deaths reached 370 thousand [4]. It was reported that 300–600 thousand individuals were affected by VTE per year in America with the number constantly growing higher [5]. Zhang et al. demonstrated VTE incidence of the newly diagnosed lung cancer in patients was as high as 13.2% in China, while prevalence of wide scale survey was scarce [6]. It was known to us that the risk factors for VTE includes age, malignancy, obesity, surgery, VTE history, immobility et al. [7–9]. The strong relationship between lung cancer and VTE was demonstrated by significant evidence that VTE incidence of lung cancer patients increased 22 times than non-cancer patients while the risk of lung cancer patients was still sevenfold higher than other malignancies [10, 11]. And how about the current status of thromboprophylaxis? A survey containing 1150 thoracic surgeons of current status thromboprophylaxis for thoracic surgery in China demonstrated that 66.96% surgeons suggested thromboprophylaxis should be administrated at first day after lung cancer resection and extended the prophylaxis after discharge, and half of the surgeons acknowledged they made the decision of prophylaxis method and duration based on their clinical experience [12]. However, American Society of Clinical Oncology Clinical Practice Guideline recommended cancer patients intended to receive major surgery should accept thromboprophylaxis before surgery and continuing for at least 7–10 days [13]. While American College of Chest Physicians Evidence-Based Clinical Practice Guidelines suggested low-molecular-weight-heparin or heparin for high risk VTE patients undergoing thoracic surgery, the start timing and duration were still lack of consensus while the recommendation for orthopedic surgery was 12 h or more preoperatively [14, 15]. Moreover, patients usually would be admitted to hospital to prepare for the surgery in China. According to the lung cancer patients usually characterized with high risk of VTE, guidelines’ recommendation and the special medical situation in our country that patients were admitted around 3 days before surgery to prepare which was different to developed countries. Combined with surgical trauma, anesthesia and malignant tumor, lung cancer patients usually featured with high VTE risk. Concerning whether conduct preoperative anticoagulation, there are no clear guidelines, no academic organization's definite instructions, and no dependable clinical experiments. Thoracic surgeons considering intraoperative bleeding and postoperative progressive hemothorax, only some of them applied postoperatively. Therefore, we innovatively designed this study to testify whether preoperative administration of LMWH for lung cancer patients was safe and feasible.

## Methods

### Patients

This trial was a prospective study and approved by the Ethics Committee of West China Hospital, Sichuan University (approval number: 20160601). Written consent was acquired from all included patients. Patients intended to receive video-assisted thoracoscopic lung cancer surgery (lobectomy and sublobar resection) under general anesthesia were included in our study from July 2017 to June 2018 in west China hospital. The inclusion criteria contained: (1) 18–75 years old without any preoperative VTEs (both VTE history and current screening); (2) patients intended for video-assisted thoracoscopic major thoracic surgery (including lobectomy and sublobarresection); (3) patients diagnosed primary lung cancer pathologically. The exclusion criteria included: (1) patients with coagulation disorders: preoperative international normalized ratio (INR) > 1.5, or blood platelet count < 50 × 109/L; (2) patients receiving any therapeutic anticoagulation preoperatively; (3) patients undergoing preoperative planned or intraoperative converted open thoracic surgery; (4) patients with severe renal or liver dysfunction; (5) patients suffered intraoperative bleeding of > 500 ml owing to vessels rupture; (6) patients rejected to continue the study at any point of the study; (7) patients suffering menstruation perioperatively. Moreover, none of the patients had received neoadjuvant chemo- and/or radiation therapy prior to surgery.

### Intervention

All the patients eligible for the study received the surgery conducted by single surgical team in Thoracic Surgery Department, West China hospital. All the patients accepted conventional three port VATS with an anterior approach with one incision and two port assist incisions under general anesthesia or minimally RATS, and one chest tube was placed after surgery. The included patients were randomly assigned to two groups by computer random system: the preoperative LMWH-administration group (PRL) and the postoperative LMWH-administration group (POL). PRL group patients received LMWH on admission to hospital with a dosage of 4000 IU percutaneous injection per day, while it would be ceased on surgical day, and administrated on postoperative day1 again until discharge. Regarding the POL group, 4000 IU LMWH per day was conducted on first postoperative day until discharge. The LMWH administration would be suspended with a drainage volume of more than 500 ml/day, and restart the thromboprophylaxis until the drainage volume decreased to less than 500 ml. The criterion for removal of chest tube was as follows: (1) the chest tube drainage volume was less than 250 ml/day (2) the chest tube demonstrated no air leak; (3) Chest radiography of the first postoperative day showed no significant abnormal signs of pleural cavity and the remaining lung. All those patients were again routinely screened for VTEs rightly after remove of chest tube.

### Observation parameters

Baseline parameters for comparison included demographic data, hematologic data, preoperative coagulation function and surgical data. Coagulation function parameters were prothrombin time (PT), activated partial thromboplastin time (APTT), fibrinogen (FIB), thrombin time (TT), and International Normalized Ratio (INR). Hematologic data mainly mean platelet count (PLT), hemoglobin value (HGB). The endpoints contained postoperative drainage data (collected by the ward nurse in our department who did not join the trial), coagulation function parameters of first and third postoperative day, reoperation rate, hematologic data, as well as data of intraoperative bleeding volume and surgical time. Drainage data included mean drainage volume per day, total drainage volume and drainage duration. The blood was extracted for three points: on admission to hospital, around 7:00 in the postoperative day 1 morning and same time at the third postoperative day.

### Statistical analysis

Statistical analysis was performed using SPSS 22.0 software (SPSS Corp., Chicago, IL, USA). Continuous variables data showed as the mean ± standard deviation were analyzed with Student’s test or Mann–Whitney U Test. As for categorical data, the chi-square or Fisher’s exact test was applied. A p-value less than 0.05 in the two- tailed test was considered significant.

## Results

**Fig. 1:**
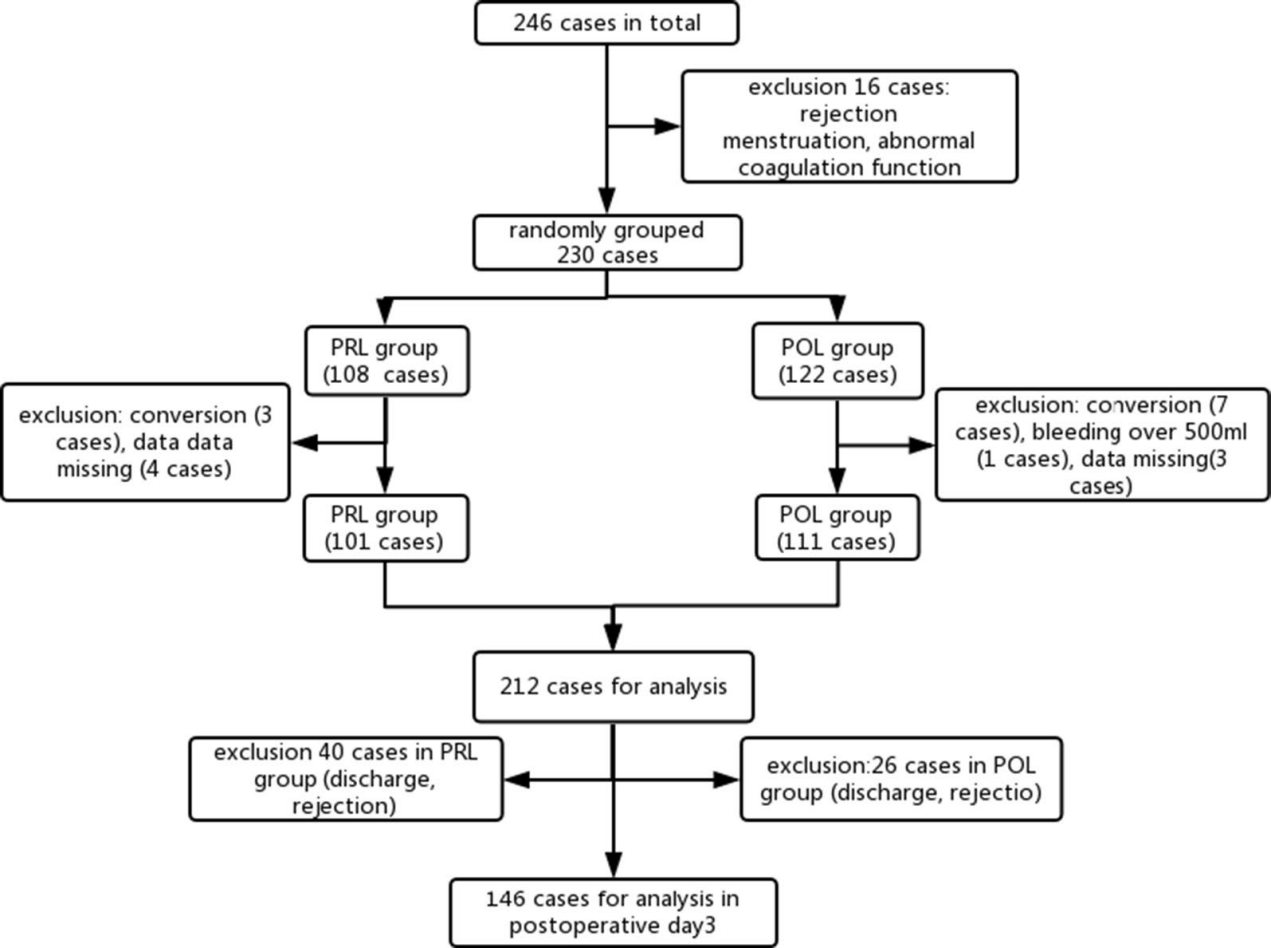
Flow chart of selecting patients in the trial

A total of 246 patients were recruited in this study From July 2017 to June 2018, 16 patients were excluded according to the exclusion criterions of rejection, abnormal coagulation function, perioperative menstruation et al. Then a population of 230 were randomly divided into two groups with 108 in PRL group and 122 in POL group respectively. Among the patients, 10 patients suffered conversion to open thoracotomy surgery due to their conditions of severe pleural adhesion or uncontrollable bleeding happened in dissecting the major vessels invaded by the tumor or the severe calcification of lymph nodes resulting in difficulty in dissection or resection. Seven patients were excluded for analysis because some data of hematologic test were missing. 1 patient from POL group suffered intraoperative artery rupture resulting in bleeding of more than 500 ml and it was excluded for analysis in this study. The flow chart was demonstrated in Fig. 1. Finally, 101 patients in PRL group and 111 patients in POL group were extracted for result analysis. And a total 0f 146 case were included to analyze the coagulation profile between the two groups in postoperative day 3.

The baseline characteristics including preoperative coagulation parameters of the two groups were comparable, except preoperative LMWH duration of PRL group was longer (Table 1).

**Table 1:** Baseline characteristics between the two groups

Characteristics	POL group (n = 111)	PRL group (n = 101)	p value
Age	55.88 ± 10.11	58.17 ± 9.99	0.100
Weight	58.12 ± 8.99	60.39 ± 9.03	0.068
Sex			
Female	70	64	0.964
Male	41	37	
Preoperative LMWH duration	0	3.98 ± 2.10	
Surgery approach			
VATS	99	83	0.144
RATS	12	18	
Surgery technique			
Lobectomy	79	70	0.767
Sublobar resection	32	31	
TNM stage			
Stage I	106	97	1.0
Stage II–IV	5	4	
Postoperative LMWH duration	3.29 ± 2.66	3.23 ± 2.32	0.860
PT	11.19 ± .62	11.31 ± 0.74	0.170
APTT	28.19 ± 3.72	28.30 ± 3.19	0.815
TT	20.08 ± 1.32	19.65 ± 2.13	0.077
FIB	2.68 ± 0.72	2.74 ± 0.78	0.523
INR	0.95 ± 0.06	0.96 ± 0.07	0.233
PLT	182.81 ± 57.51	192.82 ± 61.90	0.224
HGB	133.50 ± 13.23	134.33 ± 13.07	0.646

No VTE occurred in these patients during the study. Regarding the endpoints demonstrated in Table 2. Drainage data including drainage duration (p = 0.165), mean drainage volume (p = 0.795) and total drainage volume (p = 0.445) were all comparable between two groups. Coagulation parameters of PT (p = 0.158), APTT (p = 0.339), TT (p = 0.402), FIB (p = 0.806) and INR (p = 0.190) also showed no significant difference on first postoperative day. Moreover, there were no difference found between intraoperative bleeding volumes (p = 0.195), postoperative hemoglobin value (p = 0.735) and platelet count (p = 0.472) on Postoperative day 1. The reoperation rate of two groups were also comparable (p = 0.606). However, the operation duration of PRL group was found to be significantly longer than the POL group (117.15 ± 41.63 vs. 104.13 ± 29.07, p = 0.008). What’s more, secondary analysis was conducted about the coagulation parameters and hematologic data on third postoperative day, 85 patients for POL group and 61 patients for PRL group were remaining for the blood test until postoperative day 3 while most of the other patients discharge and a part patient blood test was ineligible. Still the parameters between the two groups did not demonstrate any significant difference shown in Table 3.

**Table 2:** Endpoints comparison between the two groups

Characteristics	POL group (n = 111)	PRL group (n = 101)	p value
PT	11.97 ± 0.86	11.82 ± 0.73	0.158
APTT	28.08 ± 4.65	27.51 ± 3.88	0.339
TT	18.73 ± 1.40	18.57 ± 1.37	0.402
FIB	3.35 ± 0.95	3.38 ± 0.68	0.806
INR	1.02 ± 0.08	1.01 ± 0.07	0.190
HGB	124.83 ± 13.02	125.44 ± 13.07	0.735
PLT	179.12 ± 53.48	184.79 ± 61.15	0.472
Drainage duration	2.70 ± 1.42	3.03 ± 1.98	0.165
Total drainage volume	569.46 ± 422.68	624.08 ± 607.95	0.445
Mean drainage volume	197.12 ± 83.23	194.09 ± 86.72	0.795
Bleeding volume	48.24 ± 57.60	39.80 ± 35.21	0.195
Operation duration	104.13 ± 29.07	117.15 ± 41.63	0.008
Reoperation			
Yes	1	2	0.606
No	110	99

**Table 3:** Coagulation function parameters of postoperative day3 between the two groups

Characteristics	POL group (n = 85)	PRL group (n = 61)	p value
PT	11.40 ± 0.75	11.85 ± 2.95	0.173
APTT	28.97 ± 3.70	28.71 ± 4.53	0.712
TT	17.25 ± 1.03	17.26 ± 0.83	0.951
FIB	5.52 ± 0.85	5.45 ± 1.01	0.633
INR	0.97 ± 0.07	0.98 ± 0.08	0.492
PLT	173.91 ± 54.90	187.95 ± 8.81	0.141
HGB	118.20 ± 14.29	120.98 ± 14.27	0.247

There were no any VTEs or blood transfusion or other major complications occurred among the enrolled patients, other than the three reoperation cases owing to pleural cavity bleeding, while no significant difference was revealed between two groups. A 53-year-old male patient came from POL group and diagnosed small cell lung cancer after radiotherapy was found the drainage appearing bright red and continuing in the ward, so the patient received exploring thoracoscopic surgery on first postoperative day finding broken end of right main bronchus artery was bleeding. Around 800 ml blood clot was removed from the cavity. The chest tube was removed on postoperative day 4 and discharged successfully without blood transfusion. The two remaining re-operation patients were extracted from PRL group. One male patient was 53 years old undergone VATS right upper lobe resection and lymph node dissection and confirmed adenocarcinoma by pathology. There was continuing a small amount of drainage out from the chest tube from first postoperative day and chest radiograph showed the pleural effusion and compress of right lung, so the patient undergone emergency exploring VATS and we found about 1100 ml mixture of blood and blood clot in the pleural cavity. Blood exudation was found consistently from medium axillary incision at fourth intercostal, then we sucked out the blood clot, made hemostasis and administrated 400 ml blood transfusion. The last one was a 63-year-old female patient with adenocarcinoma received VATS left upper lobectomy, she was characterized with low blood pressure, pleural effusion and significant decrease of hemoglobin value at the third postoperative day. Therefore, she received exploring VATS clear approximately 1500 ml blood and effusion out. Consequently, we found continuous blood exudation from pleural dome which was from pleural adhesion-cutting edge. The two patients recovered successfully and discharge soon. We thought what’s should be responsible for the three case was mainly incomplete hemostasis, administration of LMWH counts less.

## Discussion

Our study revealed that preoperative start LMWH for thromboprophylaxis of lung cancer surgery was did not influence patients regarding aspects of coagulation function, postoperative drainage, reoperation rate and bleeding volume et al. Only the operation duration of PRL group was prolonged about 13 min compared POL group with a statistically significant difference. According to our clinical practice experience, what should be mainly responsible for the result were duration for dissecting pleural cavity adhesion, lymph nodes, even the vessels and bronchus. What’s more, we thought a difference of 13 min in major thoracic surgeries could means little actually. As for the three reoperation cases, intraoperative complete hemostasis may decrease the bleeding-related events with higher chance, instead of abandoning administration of LMWH. Regarding the intraoperative bleeding volume, it was interesting that mean bleeding volume of PRL group was less than POL group (39.80 vs. 48.24 ml) without no significant difference, we considered bleeding factors such as cutting chest incision, dissecting adhesion or lymph nodes may contributed more than the result brought by preoperative administration of LMWH, which reflected the safety profile of preoperative start of LMWH in another perspective.

So far, the thromboprophylaxis efficacy of LMWH was admitted and recommended in many guidelines, but such as American Society of Clinical Oncology guideline only gave widely suggestion without specific administration point and dosage resulting in low application in clinical practice. Besides, thoracic surgeons mostly did not conduct sufficient VTE prophylaxis owing to fear of perioperative bleeding. Hence, it was urgently needed to conduct this study to testify the safety profile and provide confidence for thoracic surgeons to administrate sufficient VTE prophylaxis. Lung cancer patients were usually associated with high risk factors including cancer-, treatment- and patient- related for VTE, thromboprophylaxis for patients during hospitalization or received surgery was merely well established to prevent VTE that result in worse prognosis and mortality for patients with completely favorable safety profile [16–19]. A randomized Phase III clinical Trial from Journal of Clinical Oncology demonstrated the VTE risk of patients newly diagnosed lung cancer of any stage and histology significantly decreased from 9.7 to 5.5% after receiving prophylactic dosage of LMWH for 24 weeks, no significant difference was revealed about the major bleeding risk while the non-major bleeding events increased in the LMWH group [20]. Papageorgiou et al. found a hypercoagulability state of localized lung adenocarcinoma patients characterized by high thrombin generation and increased concentration of phosphatidylserin expressing platelet derivedmicroparticles expressing, then tumor resection and LMWH could decrease the hypercoagulable state by inhibiting thrombin generation [21]. Christensen et al. also conducted a randomized controlled trial to demonstrate that preoperative 12 h administration of LMWH until discharge did not alter coagulation profile for patients undergoing video-assisted thoracoscopic lobectomy [22]. Xu et al. also considered clinical efficacy of preoperative 12 h administration of LMWH equals to postoperative thromboprophylaxis [23]. However, what is interesting is the opposite voice, Attaran et al. randomly assigned 60 patients to receive perioperative LMWH once or twice per day and concluded not all lung cancer patients are in hypercoagulable state while the real hypercoagulable patients with careful screening should receive sufficient thromboprophylaxis [24]. Inclusion of benign disease patients, protocol of LMWH administration and stage of tumor possibly resulted in the different conclusion. Besides, a study containing 31 patients without receiving thromboprophylaxis measured perioperative coagulation state by standard coagulation tests and rotational thromboelastometry and concluded VATS surgery for lung cancer showed little influence on coagulation profile while some coagulable variables were affected actually [25]. This conclusion was completely different from routine view of cancer surgery patients characterized with high VTE risk. The small study [25] was only extracted from another big trial which could bring in selection bias and there was lack of direct comparison of administration of LMWH. Moreover, the conclusions may be resulted from the extremely small sample in those studies.

Rather than thromboprophylaxis and VTE treatment, the long-term treatment outcome of LMWH attracted growing concern. The potential mechanism of survival benefit from LMWH including Anti-proliferative actions of anticoagulants or anti-metastasis which including inhibition of microvascular growth or reduction of Epithelial-mesenchymal transition et al. [26]. Besides, LMWH decreased VTE-related morbidity and mortality [27]. A systematically review involving 952 lung cancer patients undergone chemotherapy demonstrated LMWH significantly improve the 1- and 2- year overall survival, meanwhile VTE incidence was significant reduction without increasing the side effects [28]. Altinbas et al. considered small cell lung cancer patients receiving chemotherapy plus LMWH could gain benefits of progression free survival (PFS) and overall survival [27, 29]. However, Gezelius et al. conducted a clinical phase III lung cancer trial revealed that addition of LMWH could not provide any survival benefit for small cell lung cancer patients [30, 31]. Other investigators made the same conclusion that LMWH did not demonstrate detectable survival advantage for non-small cell lung cancer patients [32, 33]. American Society of Clinical Oncology Clinical Practice Guideline did not recommend LMWH for extending survival of cancer patients [13]. Whatever, the relationship administration of LMWH and survival benefit will keep controversial for a period of time, needing more evidence to justify the controversy.

What’s more, outpatients’ VTE thromboprophylaxis is a common issue, which is difficult to monitor efficacy and conduct individual prophylaxis, then most ambulatory patients did not receive proper thromboprophylaxis even though the incidence of VTE was negligible among them. At present, clinical practice guidelines for outpatient were scarce. Elena et al. found malignancy type and VTE history were the most valuable factors for clinicians making thromboprophylaxis decision and recommend conducting prophylaxis for ambulatory patients based on individual assessment [34]. Alexander et al. demonstrated a high VTE incidence of lung cancer outpatients and majority (83%) of thromboembolic events occurred in the ambulatory care setting, that’s why ambulatory setting patients related thromboprophylaxis protocol was urgently needed [35]. A meta-analysis concluded that prophylaxis with LMWH decreased VTE occurrence of outpatients diagnosed lung cancer without significant increase of bleeding risk improving mortality [36]. However, a real world analysis revealed cancerous outpatients undergoing chemotherapy were associated with both VTE and bleeding events, it was meaning the proper assessment for outpatients’ VTE risk and exact prophylaxis was as crucial as each other [37]. As for the risk assessment model (RAM) for VTE, Caprini RAM was preferred for surgical patients and general hospitals [38]. Briefly, appropriate clinical practice protocol of thromboprophylaxis for outpatients needed further multicenter prospective study to help to establish.

Several limitations did exist in this clinical trial. First of all, a relatively small sample may minimize the evidence grade of the result. Besides, the postoperative blood test was conducted in the morning around 7 o’clock but not a specific point from the point of LMWH administration. Moreover, there were no VTEs occurring in both groups which was less convincing for preoperative start of LMWH providing sufficient thromboprophylaxis. And this study mainly explored the situation for lung cancer patients in early stage. On the other side, three case of the two groups suffered reoperation for exploring pleural cavity and making hemostasis, it seems that we should review the necessity of conducting LMWH for every lung cancer patient again. At last, this trail was just conducted in Chinese patients that the result could not be expanded to cover other races.

## Conclusion

Preoperative administration of LMWH for minimally invasive lung cancer surgery patients did demonstrate no significant effects on coagulation-related events compared with postoperative start of LMWH in this trial. The method is safe and feasible for lung cancer patients preparing to receive minimally invasive surgery. Absolutely, multicenter, prospective, randomized controlled trials are urgently needed to provide more strong evidence in the future.

## Data Availability

The datasets of this study will become available from the corresponding author on reasonable request.

## Abbreviations

LMWH: Low-molecular-weight-heparin
PRL: Preoperative Low-molecular-weight-heparin group
POL: Postoperative Low-molecular-weight-heparin group
VTE: Venous thromboembolism
DVT: Deep venous thrombosis
PE: Pulmonary embolism
VATS: Video-assisted thoracoscopic surgery
RATS: Robot-assisted thoracoscopic surgery
PT: Prothrombin time
APTT: Activated partial thromboplastin time
FIB: Fibrinogen
TT: Thrombin time
INR: International normalized ratio
PLT: Platelet count
HGB: Hemoglobin value
